# Multidimensional insights into the biodiversity of *Streptomyces* in soils of China: a pilot study

**DOI:** 10.1128/spectrum.01692-24

**Published:** 2025-04-02

**Authors:** Ziguang Deng, Wei Yang, Tongtong Lin, Yaohai Wang, Xiaojing Hua, Xiaoyu Jiang, Junhao Chen, Dan Liu, Zhiqiang Ye, Yu Zhang, Michael Lynch, Hongan Long, Jiao Pan

**Affiliations:** 1 Key Laboratory of Evolution & Marine Biodiversity (Ministry of Education) and Institute of Evolution & Marine Biodiversity, Ocean University of China, Qingdao, Shandong Province, China; 2 Laboratory for Marine Biology and Biotechnology, Qingdao Marine Science and Technology Center, Qingdao, Shandong Province, China; 3 School of Life Sciences, Central China Normal University 12446 , Wuhan, Hubei Province, China; 4 Biodesign Center for Mechanisms of Evolution, Arizona State University, Tempe, Arizona, USA; University of Mississippi, University, Mississippi, USA

**Keywords:** *Streptomyces*, biodiversity, genetic diversity, metabolic gene clusters, evolution

## Abstract

*Streptomyces,* a diverse group of filamentous bacteria found predominantly in soil, play a crucial role in nutrient cycling and produce many valuable secondary metabolites for the pharmaceutical industry. In this pilot study, we collected 19 soil samples from 14 provinces in China to preliminarily investigate the biodiversity and genetic structure of *Streptomyces* in soils of China from different dimensions, using recently developed cost-efficient amplicon and whole-genome library preparation methods. Amplicon analysis showed that Actinobacteria were among the most abundant bacteria, with 0.3% of amplicon sequence variants (ASVs) belonging to *Streptomyces*. Meanwhile, we successfully isolated 136 *Streptomyces* natural strains and assembled their genomes, including 26 previously unreported species, underscoring the need for further exploration of soil *Streptomyces* in China. Population genetics analysis revealed that homologous recombination may primarily drive the extensive genetic diversity observed in *Streptomyces*, as well as a complex population structure. Complementing this, pan-genome analysis shed light on gene diversity within *Streptomyces* and led to the discovery of rare genes, further emphasizing the vast genetic diversity of this genus. Additionally, multiple metabolic gene clusters were found in these *Streptomyces* strains, as well as some potentially unique or uncommon ones were found. These findings not only highlight the biological and metabolic diversity of *Streptomyces* but also provide a technical framework for future studies on the global biodiversity and evolution of this genus.

**IMPORTANCE:**

*Streptomyces*, a prominent group of Actinobacteria, holds significant importance in ecosystems and biotechnology due to their diverse array of metabolic products. However, research on the biodiversity of soil *Streptomyces* across extensive geographical scales in China has been limited, and their genetic diversity has rarely been evaluated using modern population genetics principles. This pilot study successfully addresses these gaps by conducting a preliminary exploration on the biodiversity of *Streptomyces* in Chinese soils from multiple perspectives, providing valuable insights for a deeper understanding of their biodiversity and a novel technical framework for future large-scale explorations of its diversity.

## INTRODUCTION

S. A. Waksman and A. T. Henrici (1) first proposed the genus *Streptomyces*, named for their resemblance to filamentous fungi. *Streptomyces* are gram-positive bacteria with diverse lifestyles that exhibit global distribution facilitated by their sporulation capability (2). They are often considered to be persistent saprophytes, which can break down and convert organic matter to maintain soil fertility (3, 4) and play a vital role in the carbon cycle (5–8). The complexity is attributed to their intricate life cycle and a high genomic G/C content that varies widely in size (6–13 Mbp), possibly enabling them to adapt to diverse environments (9–11). There are now 722 sub-taxa of *Streptomyces* with valid published and correct names (accessed on 22 March 2024 http://www.bacterio.net/streptomyces.html), reflecting their rich species diversity.

*Streptomyces* are widely studied because of their abundant secondary-metabolite biosynthetic-gene-clusters (SM-BGCs) that may encode natural products, such as antibiotics and anticancer compounds (12). These bioactive compounds produced by *Streptomyces* have greatly contributed to the pharmaceutical industry (13). Approximately two-thirds of commercially available antibiotics have been developed and manufactured by *Streptomyces* (14), thus making significant contributions to human health. Furthermore, through their influence on the synthesis of plant growth hormones, *Streptomyces* offer plants a buffer against various environmental stresses, promoting their healthy development (15). *Streptomyces* reinforce the stability and biodiversity of soil ecosystems by their intricate interactions with other soil microbes (16, 17). Therefore, studying the biodiversity of *Streptomyces* and obtaining various natural strains from soil lay the foundation for further research on their potential applications and ecological functions.

In recent years, numerous studies have explored the biodiversity of *Streptomyces* with diverse research methods. Traditionally, isolates are selectively obtained through culture-dependent methods, followed by identifying diverse characteristics and verifying rich metabolic-product functionality in *Streptomyces* based on morphological features, biochemical characteristics, growth behavior, carbon/nitrogen utilization, antibiotic sensitivity, and specific activities (18–21). However, this approach often lacks genomic and genetic information on strains and is unsuitable for large-scale microbial biodiversity studies. Molecular methods, such as whole-genome sequencing (WGS), multi-locus sequence typing, and metagenomics, can overcome these issues. However, studies on the genomes and genetic diversity of *Streptomyces* are typically limited to small-scale research (22). Besides cost considerations, many studies focus only on genetic differentiation or adaptive evolution mechanisms within a single species or closely related species (23, 24), rather than on the broad genetic and secondary metabolite functional diversity across various *Streptomyces* species, which could lay the groundwork for potential future applications.

**Fig 1:**
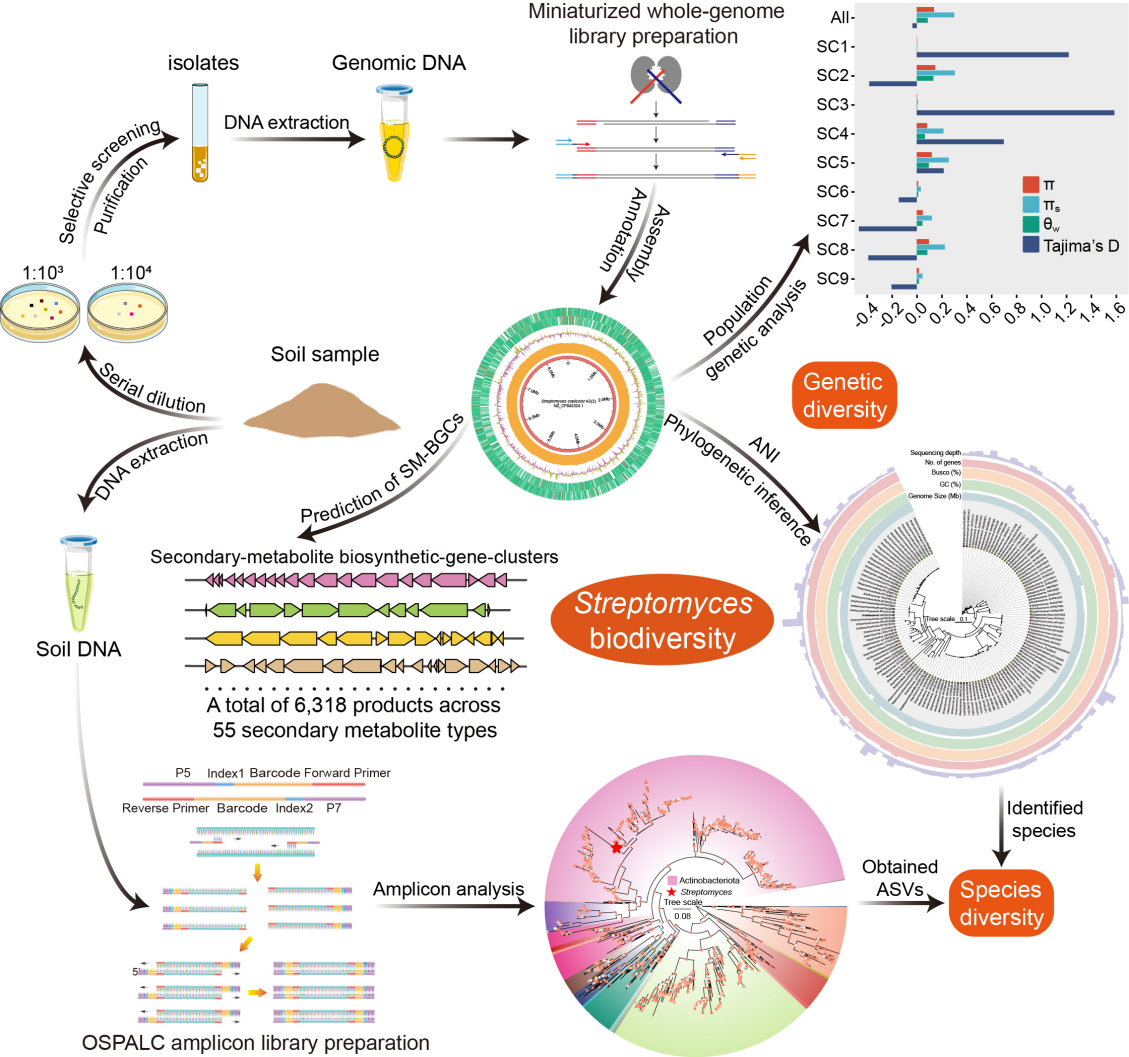
A multidimensional survey of *Streptomyces* diversity across 19 soil samples recently collected from mainland China. Amplicon (25), whole-genome (23), and culture-dependent methods were all used to analyze the species and genetic diversity of *Streptomyces*.

In this preliminary study, we initiated a survey of *Streptomyces* diversity across 19 soil samples recently collected from mainland China (Fig. 1). Simultaneously, strain isolation and culturing methods facilitated the isolation of 136 *Streptomyces* isolates from these samples. We used in-lab developed library preparation methods to obtain sample sequences at a low cost, enabling analysis of *Streptomyces* biodiversity in soil samples from multiple dimensions. The rigorous and multifaceted approach, including species identification, whole-genome sequencing, genome assembly and annotation, population genetic analysis, and pan-genome analysis, showed high species and genetic diversity and a broad array of secondary metabolites. By employing the integrative, multidimensional analysis strategy mentioned above, we can efficiently and comprehensively explore the biodiversity of soil-derived *Streptomyces*, forming a solid foundation for more expansive investigations into their biodiversity and evolution, as well as facilitating further exploration of gene clusters and drug development.

## MATERIALS AND METHODS

### Sample collection and isolation

We collected soil samples from 19 sites across China, covering a distance of over 2,000 km in both north–south and east–west directions (Fig. S1; Table S1). The majority of samples were collected from 0 to 5 cm top soil from March 2020 to May 2021. We stored these samples in a freezer at −80°C immediately after collection. To isolate *Streptomyces*, 1 g of soil was diluted in sterilized Ultrapure water at a ratio of 1:10 (wt/vol) and vigorously mixed at room temperature for 10 minutes. Following a 7-minute heating in a 65°C water bath, we further diluted the soil extract at 1:10^3^ or 1:10^4^ (vol/vol) ratios. About 100 µL of each diluted soil extract was inoculated on five modified 2216E solid medium plates, which included 20 mg/L nalidixic acid (Thermo Fisher Scientific, Cat. No.: 389–08-2) (26, 27). After 4 days of incubation at 28°C, we then selected single Actinomycete colonies. We stored the cell lines as spore suspensions in 20% glycerol after three consecutive streaking on agar plates without antibiotics.

### DNA extraction

Soil samples’ DNA for amplicon sequencing was extracted using the FastDNA Spin Kit for soil DNA extraction (MP Biomedicals, Cat. No.: 6560200; 0.5 g soil for each sample). We also cultured isolated Actinomycete cell lines in 5 mL of the modified 2216E liquid medium at 28°C with shaking for 5 days. Cultured cells were briefly washed twice by centrifugation at 16,000 rpm with 1 × PBS (pH = 7.4). Subsequently, we suspended the washed cells in 1.5 µL lysozyme solution (100 mg/mL) and incubated them at 37°C overnight. We then extracted high-quality genomic DNA using the MasterPure Complete DNA & RNA Purification Kit (Lucigen/Epicentre, Cat. No.: MC85200).

### 16S rRNA gene amplification, amplicon, and whole-genome library construction/sequencing

The genomic DNA of the isolates was amplified using primers 8F (5′-AGAGTTTGATCCTGGCTCAG-3′) and 1492R (5′-CGGTTACCTTGTTACGACTT-3′) to target the 16S rRNA gene for Sanger sequencing (28). We used a 15 µL PCR system, which included 7.5 µL of 2 × Phanta Flash Master Mix (Dye Plus), 0.5 µL of the 10 µM forward primer, 0.5 µL of the 10 µM reverse primer, 5.5 µL of nuclease-free H_2_O, and 10 ng of the template DNA. The PCR conditions involved an initial denaturation at 98°C for 5 minutes, followed by 18 cycles of 98°C for 30 seconds, 55°C for 30 seconds, and 72°C for 2 minutes, with a final extension at 72°C for 7 minutes. Finally, we Sanger-sequenced the PCR products at Tsingke Biotechnology Co., Ltd. We trimmed and assembled the sequences using SeqMan (29). We performed genus-level validation using NCBI BLAST (accessed on 22 March 2024 https://blast.ncbi.nlm.nih.gov/Blast.cgi) (30).

Out of the 19 soil samples, we successfully prepared 18 amplicon libraries using the highly cost-efficient OSPALC protocol recently developed by our lab (25) (~7% of the charge by service providers per sample library). The library amplification used a regular PCR system and long primers containing universal short primers targeting the 16S rRNA gene V3–V4 region (31) and the sequencing adapters. We sequenced the libraries using the PE250 mode on an Illumina NovaSeq 6000 sequencer.

For the isolated *Streptomyces*, we prepared whole-genome libraries with the Vazyme TruePrep DNA Library Prep Kit V2 (Vazyme, Cat. No.: TD501-01) for Illumina, following a recent miniaturized protocol developed by our lab (23) (~1/6 of the charge by service providers per sample library). Illumina PE150 sequencing was done at Berry Genomics, Beijing, and Beijing Novogene Genomics Technology Co., Ltd. (Beijing, China).

### Amplicon analysis

We performed amplicon analysis using QIIME2 v2023.7 (32). We merged raw reads and subsequently filtered them using qiime vsearch (33) and qiime q-score, respectively. Feature classification was performed with qiime deblur (34). We used the Silva database (release 138, 99% OTUs full-length sequences) (35–39), specific to the 16S rRNA gene V3–V4 region. Additionally, we aligned feature reads using QIIME alignment MAFFT, and phylogenetic trees were constructed using FastTree2 (40), followed by visualization of all phylogenetic analysis results with ChiPlot (41).

### Genome assembly, annotation, and phylogenomic inference

For the 136 isolates with genomes successfully sequenced (sequencing details are in Table S1), we filtered raw reads with fastp v0.23.4 (42) for quality control and subsequently performed *de novo* assembling using Unicycler v0.5.0 (43). The assemblies were evaluated through QUAST v5.0.2 (44) and BUSCO v5.4.0 (45), and Table S1 lists all genome-related information. We annotated the genomes using Prokka v1.14.5 (46) with default parameters, and through the annotation files, we extracted the sequences of the *rpoB* gene from the genomes using shell scripts for species-level identification. Then, we calculated the pairwise genome-wide ANI using PYANI v0.2.11 (47).

We obtained single-copy core genes, defined as orthologous groups (OGs) with a single copy in each genome, from OrthoFinder v2.5.4 (48) analysis results. Each single-copy core OG was aligned with MUSCLE v3.8.1551 (49) at the amino acid level and then nucleotide level through PAL2NAL v14 (50). We trimmed poorly aligned regions using Gblocks v0.91b (51), with -t = c. The resulting alignments were concatenated with a custom Python script to obtain core genome sequences. We reconstructed a phylogenetic tree based on the concatenated single-copy core genes using IQ-TREE v2.2.3 (52) under the general time reversal model and visualized it using ChiPlot (41).

### Pan-genome analysis and prediction of secondary metabolite biosynthetic gene clusters

OGs in the pan-genome were identified using MCL algorithms (van Dongen and Abreu-Goodger, 2012) (BLASTP E-value cutoff = 1e-5; inflation value = 1.5) implemented in OrthoFinder v2.5.4 (48), and genome statistics within the pan-genome, derived from the analysis results of OrthoFinder v2.5.4, were visualized using PanGP v1.0.1 (53) with the distance guide sampling algorithm.

Furthermore, we used Python scripts written in Snakemake v8.0.1 (54) to predict SM-BGCs for a batch of 136 isolated soil *Streptomyces* genomes. The scripts employed antiSMASH v7.0 (55) for prediction and simultaneously recorded the counts of distinct secondary metabolite types for each genome.

### Genetic diversity and homologous recombination analysis

We performed haplotype identification using Haploview v4.2 (56) and determined nucleotide diversity (π), nucleotide diversity at silent sites (π_s_), the number of segregating sites (S), Tajima’s D, and Watterson’s theta per site (θ_w_) with DnaSP v6 (57). We determined the pairwise homoplasy index (Phi) statistic using PhiPack v1.1 (58) and evaluated statistical significance under a null hypothesis of no recombination. The calculation of the fixation index (*F_st_*) from single-copy core genes was done with VCFtools v0.1.16 (59).

FastGEAR (60) identifies the population genetic structure of an alignment in question and detects recombination between the inferred lineages as well as from external origins. Specifically, we used fastGEAR to identify non-ancestral and ancestral recombination events by analyzing the alignments of concatenated single-copy core genes across all 136 isolates, which allowed the reconstruction of recombination events among the lineages of *Streptomyces* and among the genetic clusters identified by BAPS v5.3 (61). We combined and plotted the output of every core gene from fastGEAR using post-processing scripts (62) and used ggplot2 (63) to visualize recent and ancestral recombination events.

## RESULTS

### Low relative abundance of *Streptomyces* revealed by amplicon analysis

We extracted DNA from soil samples collected across 19 sites in China and sequenced their 16S rRNA gene V3–V4 regions with the Illumina NovaSeq 6000 PE250 mode. Successful sequencing was achieved for 18 of the samples (Fig. S1). On the raw sequences, we applied de-multiplexing, paired-end read merging, quality filtering, denoising, and chimera removal, which led to a total of 243,803 high-quality sequences, defined as “features” for subsequent amplicon analysis. On average, each soil sample contained 10,765 ± 2,367.3 features.

**Fig 2:**
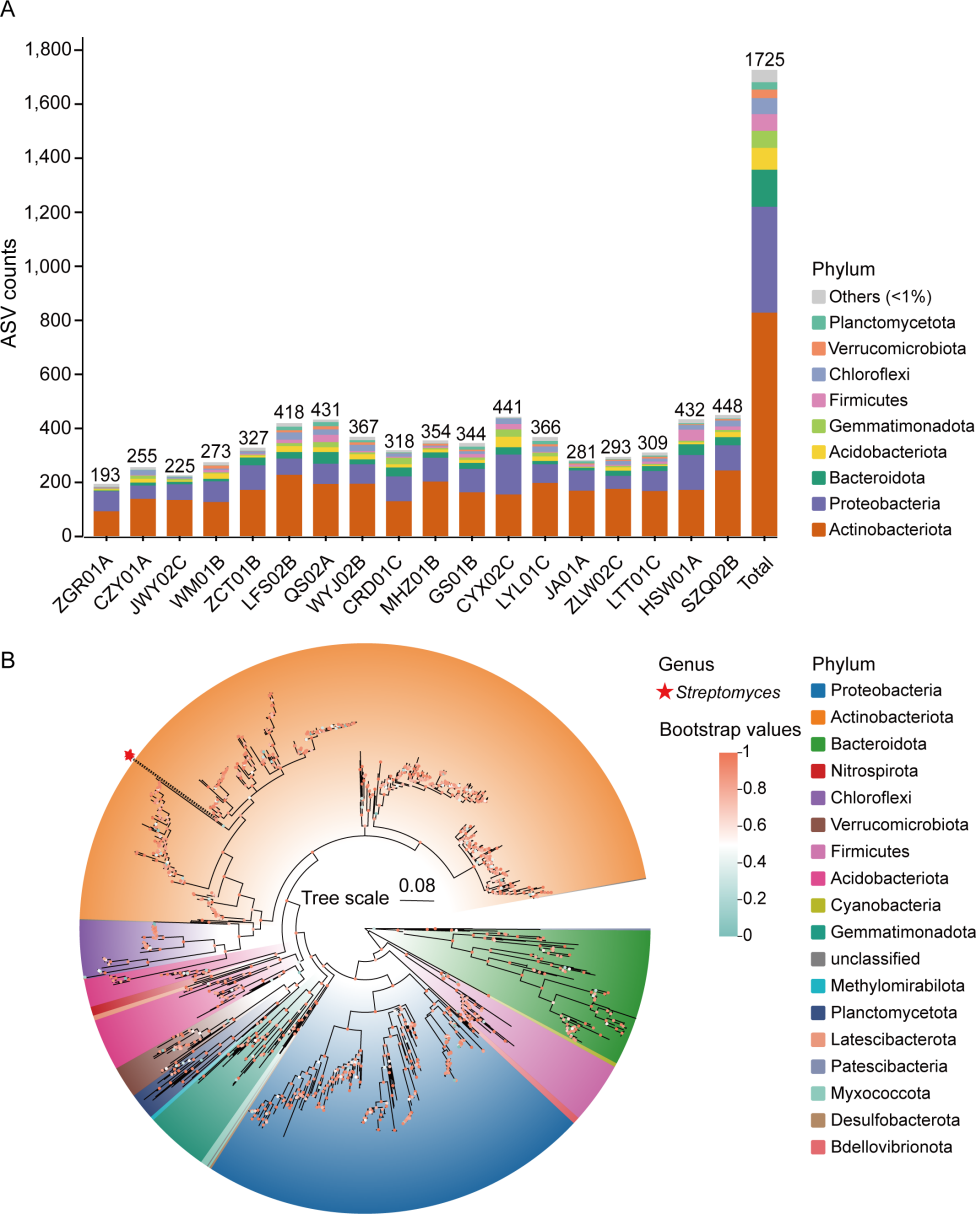
Numerical and taxonomic amplicon sequence variants (ASVs) recovered by OSPALC amplicon sequencing on the soil samples. (**A**) ASV counts and bacterial phylum distribution within each soil sample. (**B**) ASV-based phylogenetic tree. The colored regions illustrate the distribution of ASVs across all detected phyla, and the inner circles at the nodes of the branches display the bootstrap values for each branch. Additionally, the stars at the ends of the branches represent *Streptomyces*.

**Fig 3:**
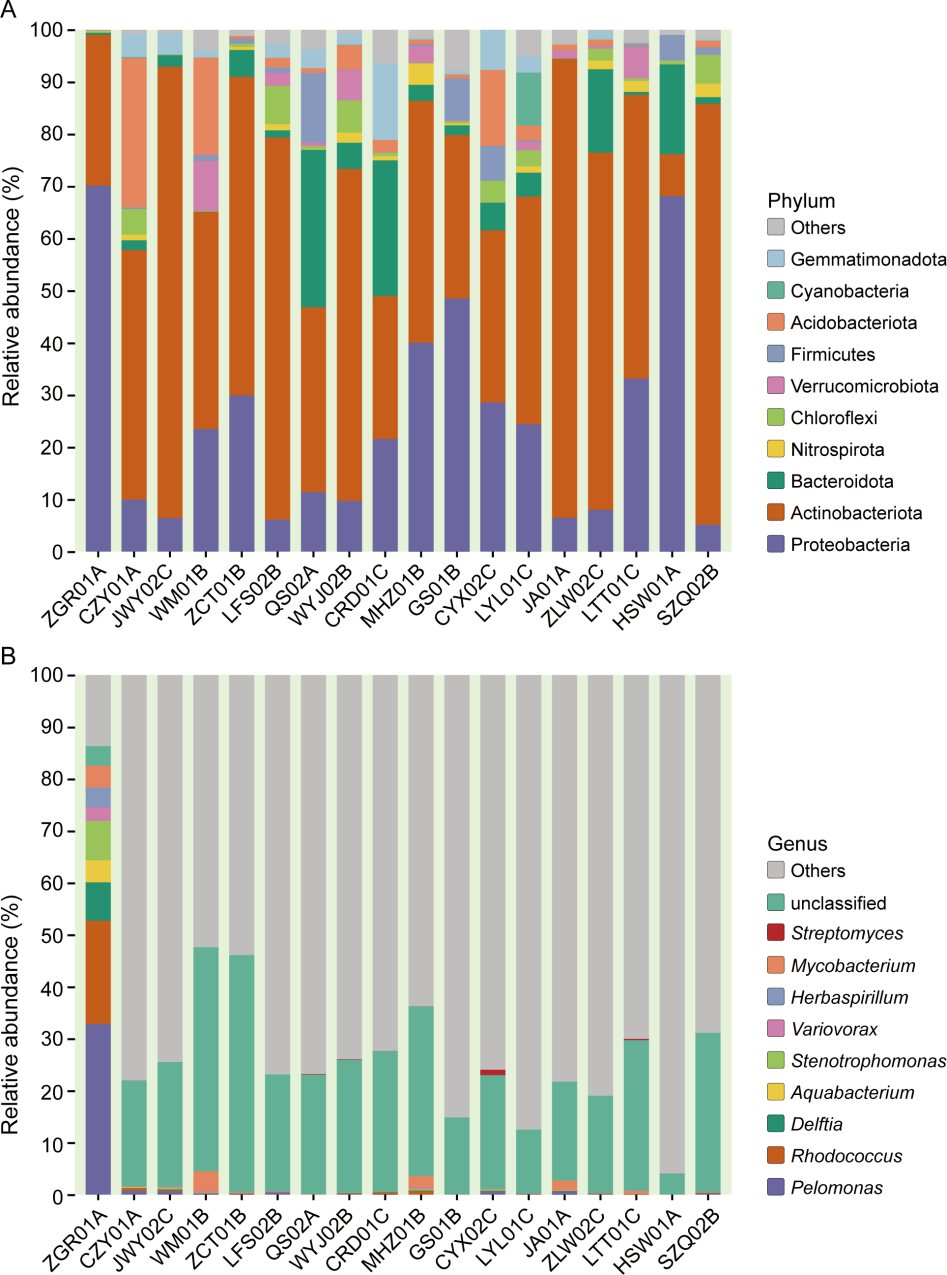
Relative compositions of bacterial communities at phylum (**A**) and genus (**B**) levels. (**A**) The bar chart depicts the relative abundance distribution of the phylum, and the top 10 taxa with the highest relative abundances are listed, along with the category “Others.” (**B**) Relative abundance distribution at the genus level, and the red bar represents *Streptomyces*.

Across the 18 successfully sequenced samples, we identified 1,725 distinct amplicon sequence variants (ASVs) and examined taxonomic classifications from phylum to genus levels of bacteria. At the phylum level, Actinobacteriota (47.94%) represented the most abundant phylum in terms of ASV diversity (Fig. 2). However, at the genus level, only five ASVs (0.29%) belonged to the *Streptomyces* species (Fig. 2B). The results were further supported by the specific taxonomic relative abundance distribution at the phylum and genus levels within each soil sample. We found that the abundance of Actinobacteriota was the highest among bacteria in each soil sample, except HSW01A (Fig. 3A) and *Streptomyces* species were exclusively found in five soil samples: QS02A (0.12%), WYJ02B (0.09%), CYX02C (1.06%), LTT01C (0.23%), and HSW01A (0.003%), with the highest abundance observed in CYX02C (Fig. 3B). The results revealed varied microbial diversity among soil samples, with Actinobacteriota dominating and effectively characterizing their biodiversity. *Streptomyces*, a genus within Actinobacteriota, is notably absent or minimally abundant in most samples. This is consistent with previous reports that *Streptomyces* are not dominant members compared with other Actinobacteria (64, 65).

### High genomic variation of the isolated *Streptomyces*

**Fig 4:**
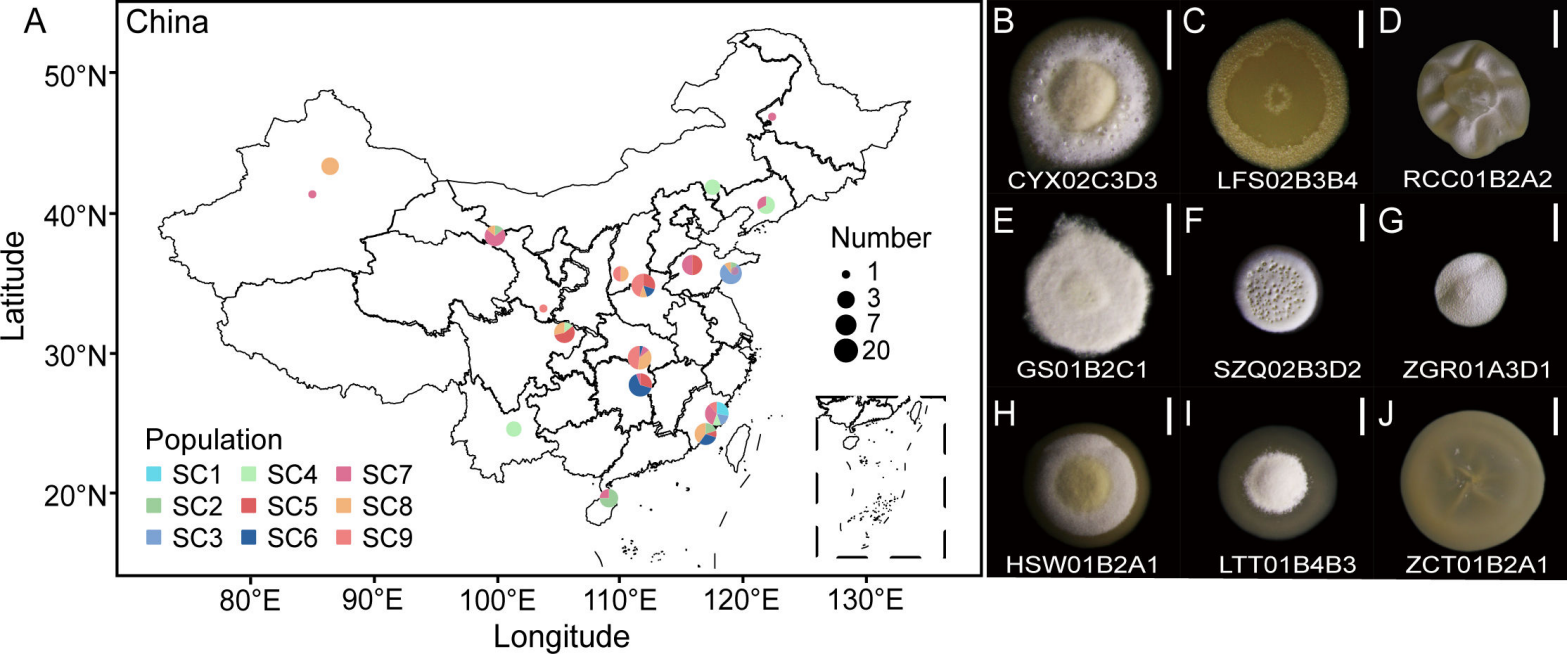
Sampling sites and photomicrographs of isolated *Streptomyces*. (A) The circle colors represent different populations across China, while the size of the circles represents the number of *Streptomyces* isolates obtained from each soil sample. The map was generated using the open-source MagicJs (https://github.com/iuvc/magicJs). (B–J) The colony morphology of nine *Streptomyces* isolates. The size of the scale bars is 0.5 mm.

In order to comprehensively and precisely investigate the biodiversity of *Streptomyces* in the soil samples, we also used a culture-dependent approach and applied a miniaturized genome-library preparation protocol that we recently developed (23). The colony morphology of *Streptomyces* is typically stable, characterized by elongated white aerial spores around the periphery and a firm surface. Colonies with these similar phenotypes were isolated, cultured, and subsequently subjected to sequencing of their 16S rRNA genes for identification. After repeating the procedures for 8 months, we eventually isolated 136 cell lines of *Streptomyces* spp. from all the soil samples initially used for amplicon analyses (Fig. 4).

The genomes of these isolated *Streptomyces* were sequenced using Illumina NovaSeq 6000 PE150, followed by assembly and annotation of the draft genomes (Table S1). The general genomic features of all isolates are highly variable, as shown in Table S1. The genome size ranges from 6.68 to 10.82 Mbp, the number of genes per genome ranges from 5,863 to 9,710, and the G/C content is 69.48% to 73.65%. Additionally, the BUSCO scores for each genome ranged from 91.3% to 100%, indicating a high level of genome completeness (45). Considering the typical prokaryotic species cutoff ANI (average nucleotide identity) value of 95% to 96% (66, 67), we categorized the isolated *Streptomyces* into 44 species. Specifically, we identified and analyzed the closest reference species from NCBI genome in terms of ANIblast for these lines and statistically compared their ANI (Table S2). Notably, only 40.44% of the lines showed an average ANI above 95% with their closest *Streptomyces* species (totaling 18), indicating that the remaining lines are potentially unreported species of *Streptomyces* and calling for future systematic research on *Streptomyces* biodiversity in China’s soil.

**Fig 5:**
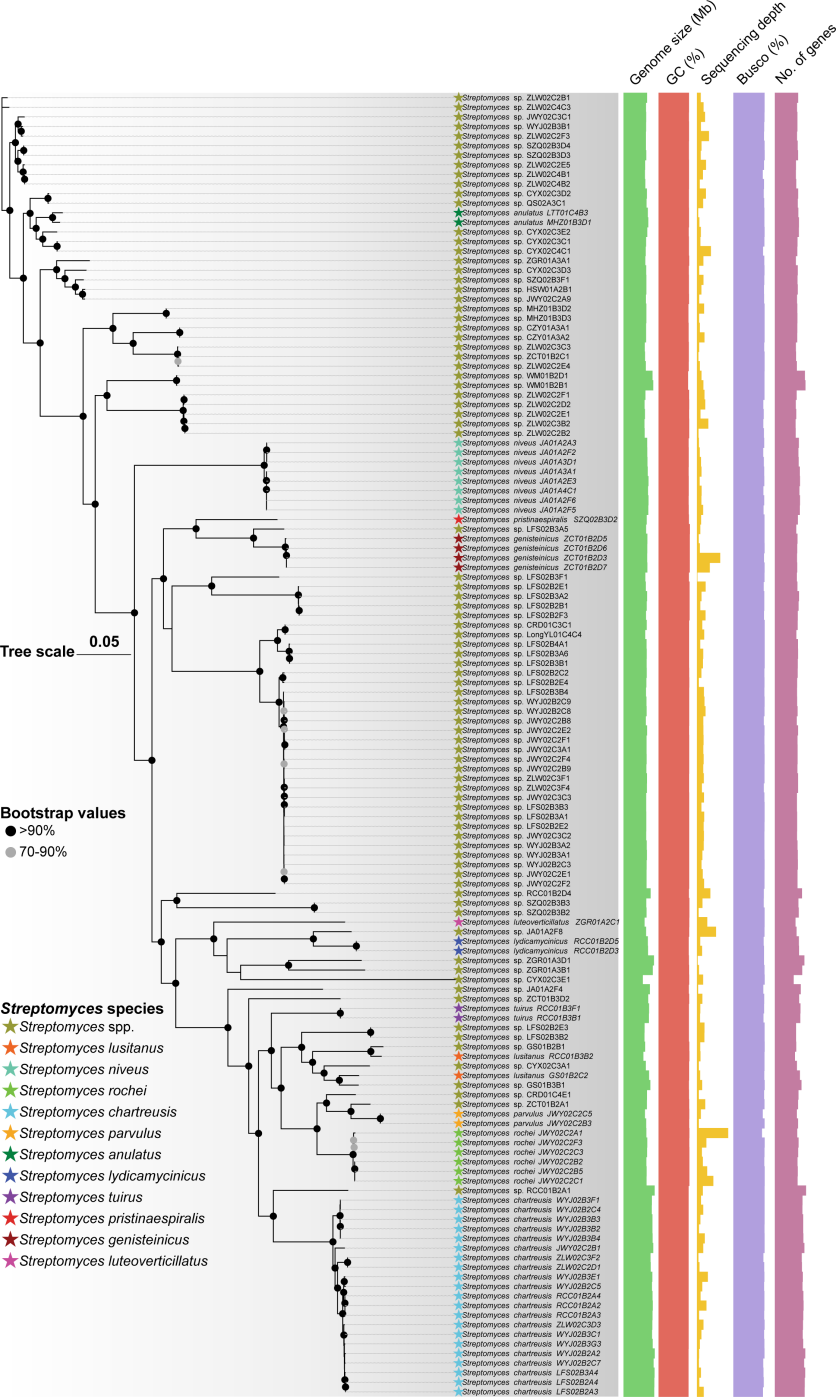
Maximum likelihood tree constructed based on concatenated single-copy core genes with the GTR +GAMMA evolution model. The scale bar represents nucleotide substitutions per site. *Mycobacterium smegmatis* (hide) is the outgroup. Nodes with bootstrap values > 70 are marked with gray circles, while those >90 with black circles. The colored stars in front of species names represent different species (Table S2). The rightmost five colored bands represent different genomic features for the isolates. The collection sites and other features for each isolate can be found in Table S1.

It was also found that the ANI varies extensively among the genomes of the 136 isolates, which ranged from 84.25% to 99.99%, and to some extent, reflects variations in their evolutionary history and high genetic diversity. To demonstrate this, we then constructed a maximum-likelihood (ML) phylogenomic tree, based on concatenated single-copy core genes (totally 166,959 bp; Fig. 4 and 5; Table S2). The clustering results of *Streptomyces* lineages in the phylogenetic tree mutually corroborated the previous species classification (Fig. 5).

### Homologous recombination events as a driver of genetic diversity in soil-derived *Streptomyces*

**Fig 6:**
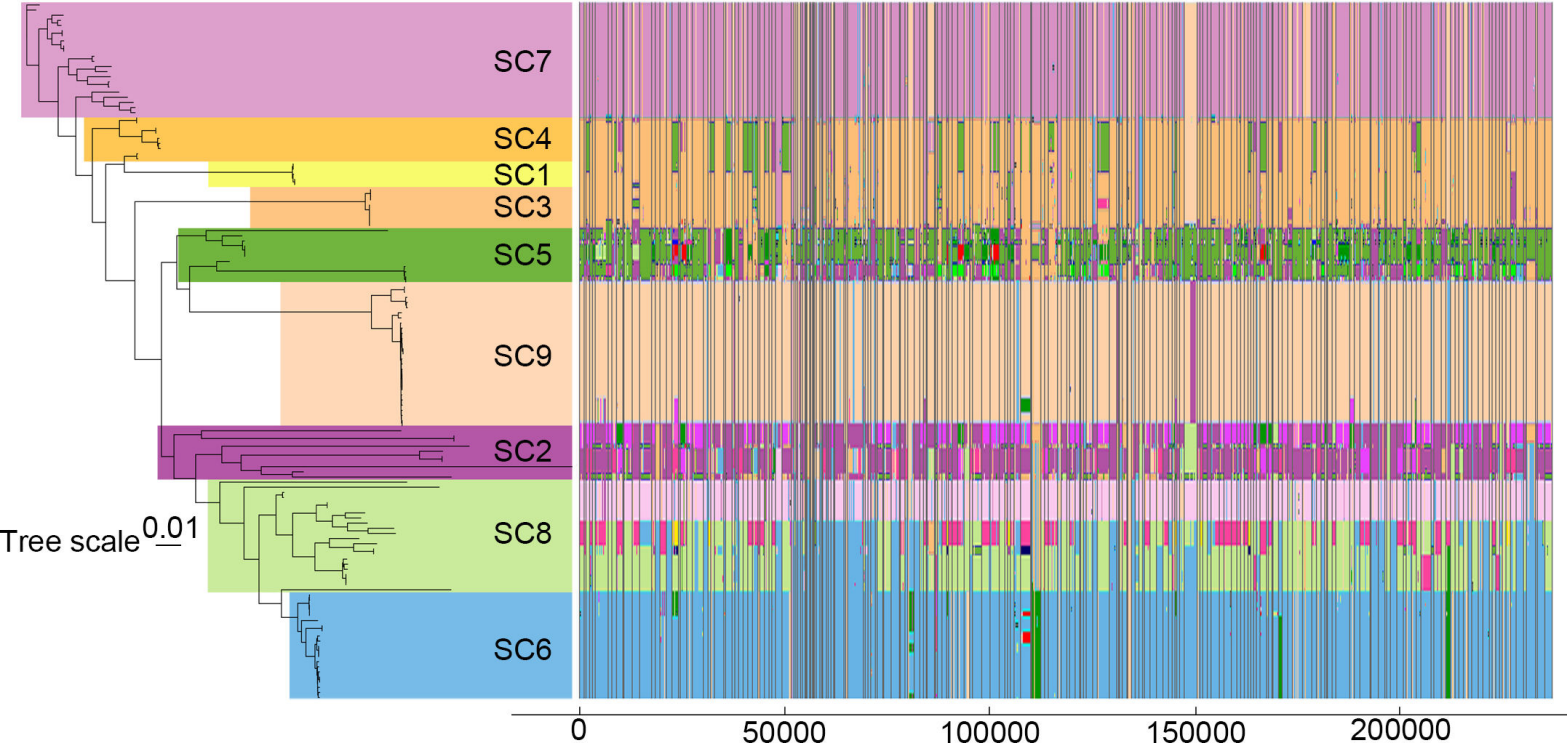
Population structure of the 136 *Streptomyces* isolates. The phylogeny and sequence clusters (SCs), represented by modules of varied colors on the left, show the single-copy core-gene-based tree with nine monophyletic clusters. The right panel shows fastGEAR output for 215 single-copy core genes, as discussed in the text. The axis at the bottom shows the coordinates of the alignment of the 215 concatenated genes in bp. The colors represent different lineages identified in the analysis (but are otherwise selected arbitrarily to be easily distinguishable). Recent and ancestral recombinations are with the color of the donor lineage. The results for different genes were obtained by running fastGEAR independently, but the lineage colors at different genes were reordered to reduce the number of colors for any single isolate across the genes. White color denotes missing data, and black color denotes recombination estimated to come from outside of any lineage in the data set.

**Fig 7:**
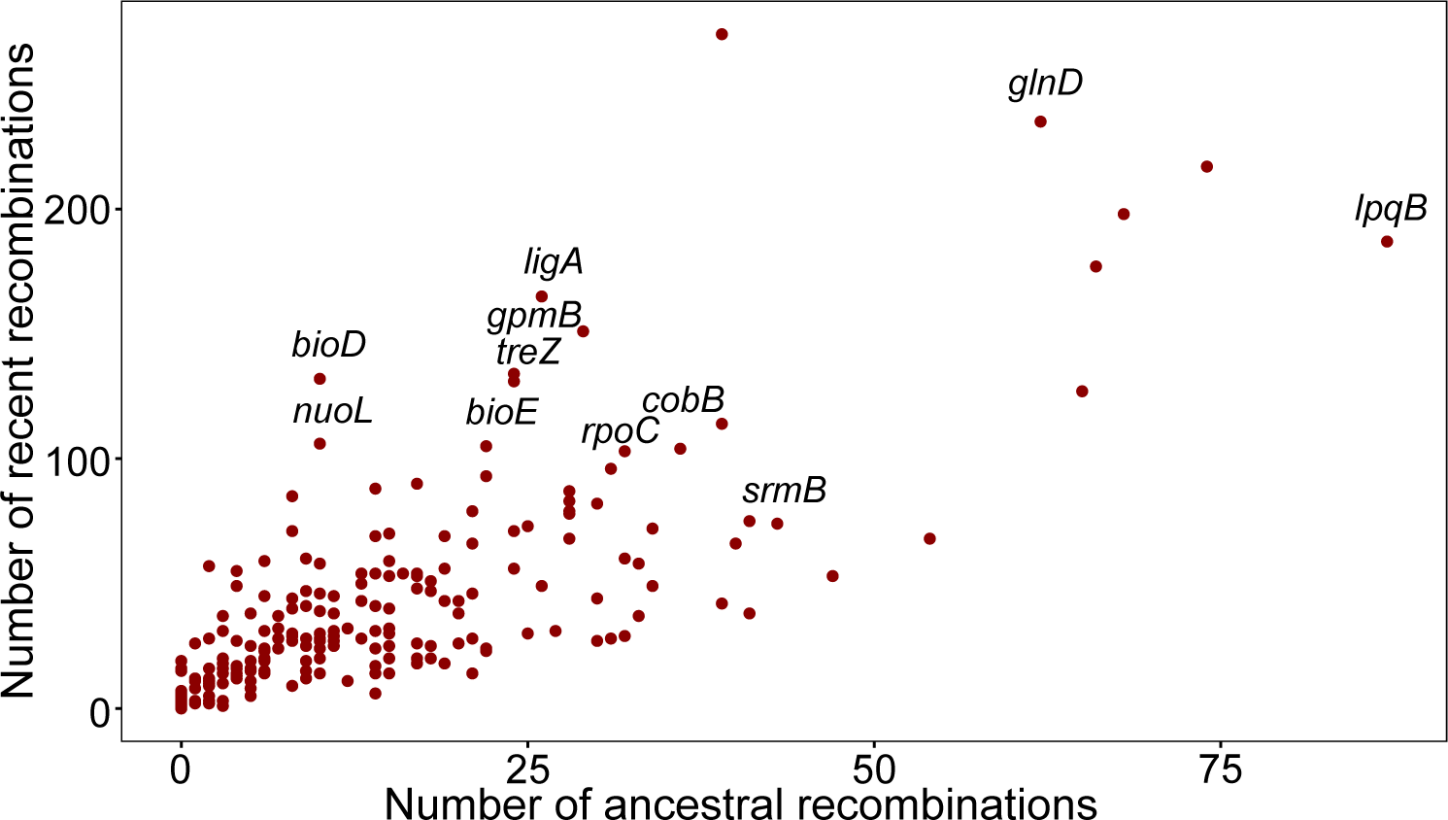
Comparing the degree of mosaicism across genes. The X-axis represents ancestral recombination event counts for each homology group, while the Y-axis represents the counts of recent recombination events in the 136 isolates detected per homology group. The names of some genes with the highest recombination events (sum of the recent and the ancestral recombinations) are labeled in the plot.

To reveal the processes generating the differentiation among *Streptomyces* isolates, we investigated the gene flow of single-copy core genes among lineages. Our results based on the concatenation of SCGs delineate the *Streptomyces* population into nine monophyletic clusters. However, the distinct clusters identified by individual SCGs do not align precisely with different lineages; instead, they tend to form “average” clusters to some extent (Fig. 6). This phenomenon possibly arises from varying degrees and locations of homologous recombination in each SCG, promoting genetic differentiation within genes and across the genome, enhancing genetic diversity. Subsequently, we quantified recent and ancestral recombination events detected in each single-copy core gene (Fig. 7). The top 20 genes with the highest occurrence of recombination events, each with the function fully annotated (Table S3), are predominantly associated with biological functions of cellular metabolism, functional regulation, and genetic information transfer. Recombination events on these essential genes may signify an increase in genetic diversity and alterations in gene function fostering genome evolution. Additionally, we calculated the pairwise homoplasy index (Phi) values for single-copy core genes within each *Streptomyces* population. The results revealed that the Phi values were statistically significant for most populations, except for SC1 and SC3 (Table S4). This evidence indicates substantial homologous recombination within the *Streptomyces* populations, driving genetic differentiation and diversity. Such results are highly consistent with those of previous explorations on genetic diversity determinants in *Streptomyces* (68, 69).

### High genetic diversity revealed by population genetics analysis on *Streptomyces* isolates

Traditional *Streptomyces* genetic diversity studies routinely focused on gene richness and allele count. This is not in line with modern population genetics, which focuses on genetic diversity metrics such as nucleotide diversity, shaped by evolutionary mechanisms such as mutation, selection, and homologous recombination (70, 71). Therefore, we performed comprehensive population-genetic analyses to accurately evaluate genetic diversity within and among populations of isolated *Streptomyces* in this study.

All *Streptomyces* isolated through culture-dependent methods were categorized into nine monophyletic clusters, as described (Fig. 4 and 6). The parameters such as number of segregating sites (*S*), per site nucleotide diversity (π), per silent site nucleotide diversity (π_s_), per site Watterson’s theta (θ_w_), and Tajima’s D are closely associated with population genetic diversity (Table 1). Generally, an *S* value in a population is usually used for quantifying the genetic variation. Large differences in *S* values between populations were observed (ranging from 677 to 61,720), reflecting substantial disparities in their levels of genetic variation and genetic diversity. The consistent trends of *S* values and θ_w_ suggest that differences in effective population size might be an important factor contributing to this variation. The significant differences between π and π_s_ (π_SC2_≈46π_SC3_; π_sSC2_≈36π_sSC3_) further indicate extensive nucleotide diversity among populations, where differences in effective population size also play a significant role. By comparing these values with those in one previous study (72), SC2 and SC5 are revealed to exhibit high genetic diversity (e.g., Min ANI < 85; π > 0.1, π_s_ > 0.25), while SC1 and SC3 show low levels of genetic diversity, probably due to the fact that each of them was isolated from a single soil sample and containing only one species (Tables S1 and S2). In contrast, other populations isolated from multiple soil samples from different locations contained multiple species, reflecting higher genetic diversity. Moreover, we calculated Tajima’s D values to assess the impact of selective pressures on genetic variation. The range of Tajima’s D values (–0.46 to 1.59) suggests diverse modes of selection in different *Streptomyces* populations, with positive values indicating positive selection and negative values indicating negative selection. The positive selection pressure is more pronounced in SC1 and SC3 (Tajima’s D > 1). Additionally, we used the Fixation Index (*F_st_*) to characterize the genetic differentiation among all *Streptomyces* populations, considering variations in the frequencies of single-copy core genes. Our findings reveal some genes with high *F_st_* values, indicating strong population structures between populations (Fig. S2).

**TABLE 1:** Information on the *Streptomyces* populations used in this study^*a*^^,^^*b*^

Population	Ave Lat	N	Min ANI	S	π	π_s_	θ_w_	Tajima’s D
All	34.43°N	136	81.11	85,031	0.1373	0.3000	0.0881	−0.0351
SC1	26.06°N	5	99.67	677	0.0020	0.0049	0.0017	1.2198
SC2	26.30°N	7	80.41	61,720	0.1483	0.3051	0.1332	−0.3813
SC3	36.11°N	8	99.43	1,241	0.0032	0.0084	0.0025	1.5861
SC4	33.38°N	9	87.14	34,034	0.0830	0.2137	0.0648	0.6983
SC5	33.83°N	14	83.39	57,324	0.1196	0.2565	0.0955	0.2164
SC6	28.44°N	21	97.54	7,732	0.0112	0.0324	0.0111	−0.1444
SC7	32.50°N	22	90.76	31,487	0.0472	0.1207	0.0448	−0.4642
SC8	32.53°N	22	85.04	57,000	0.0970	0.2255	0.0844	−0.3892
SC9	31.43°N	28	96.81	12,614	0.0165	0.0450	0.0166	−0.2025

^*a*^ Single-copy core genes were concatenated for 5–136 *Streptomyces* isolates from each population. Summary statistics for concatenated nucleotide sequences were determined as described in the *Methods* section. Average latitude (Ave Lat) was calculated using the total number of isolates per population and is shown in Table S1.

^*b*^ Ave Lat, average latitude of populations; N, number of *Streptomyces* isolates for each population; Min ANI, minimum percent pairwise average nucleotide identity (ANI) within population isolates; *S*, number of segregating sites; π, per site nucleotide diversity; π_s_, per silent site nucleotide diversity; θ_w_, per site Watterson’s theta (*S*/a_n_; *S*, number of segregating sites, a_n_, an adjustment factor related to the effective population size); Tajima’s D, Tajima’s test ((π-θ_w_)/*S*, the parameters explained as before).

### Extreme gene divergence between strains revealed by the pan-genome

**Fig 8:**
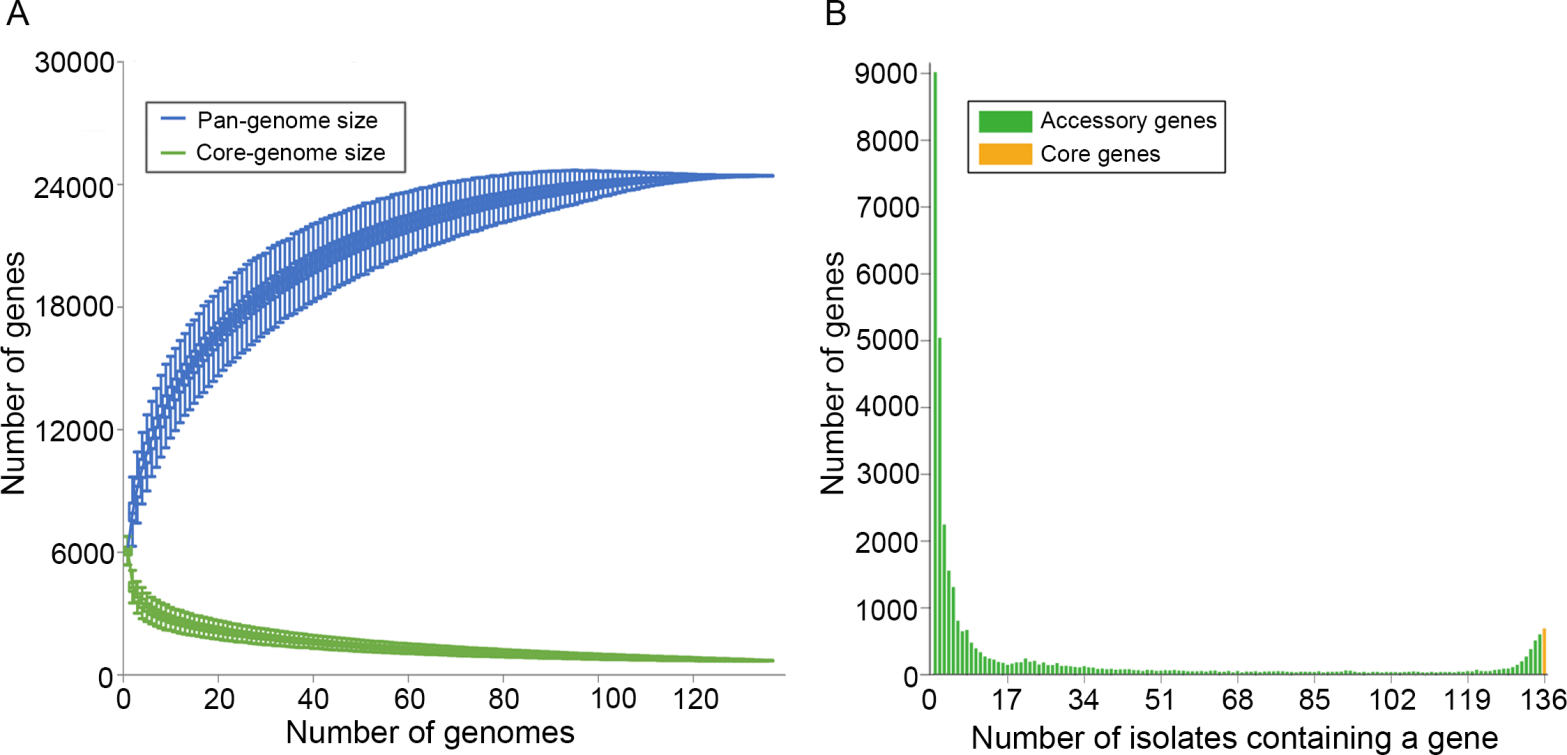
Pan-genome, core genome, and accessory genome features of the isolated *Streptomyces*. (**A**) The sizes of the core and pan-genomes are illustrated concerning the number of genomes added to the gene pool. Box plots represent the 25th and 75th percentiles, with medians as horizontal lines. Whiskers indicate the lowest and highest values within 1.5 times the interquartile range (IQR) from the first and third quartiles. The pan-genome curve is modeled by the power-law regression (*y_pan_* = *A_pan_ x^Bpan^ +C_pan_*), with r^2^ = 0.991, *A_pan_* = 370,895 ± 393.71, *B_pan_* = 0.01, and *C_pan_* = −366,289 ± 410.56. Here, *B_pan_* corresponds to the parameter γ, and α (= 1-γ) <1 signifies that the pan-genome does not converge to a constant with additional genome sampling. The core genome curve is fitted with the exponential curve model (*y_core_* = *A_core_ e^Bcore.x^ +C_core_*), with r^2^ = 0.922, *A_core_* = 3,352.15 ± 6.88, *B_core_* = −0.03, and *C_core_* = 849.28 ± 0.63. (**B**) Gene distribution across the isolated *Streptomyces* is presented.

A pan-genome was assembled using the genomes of the 136 *Streptomyces* isolates assembled in this study. The pan-genome contains 24,422 genes and 682 OGs (orthologous genes), including 364 single-copy core genes (Fig. 8A). The core genome constituted only 7.02% to 11.63% of the gene content of each strain. Besides, the size of the pan-genome and core-genome of *Streptomyces* approached a constant as more genomes were sampled (α ≈ 1, estimated by power-law regression), indicating that their genetic diversity reached saturation, and most (17,831) accessory genes were unique or rare (existing in one to four isolates) (Fig. 8B). The results revealed large genetic divergence among genomes of *Streptomyces*.

**Fig 9:**
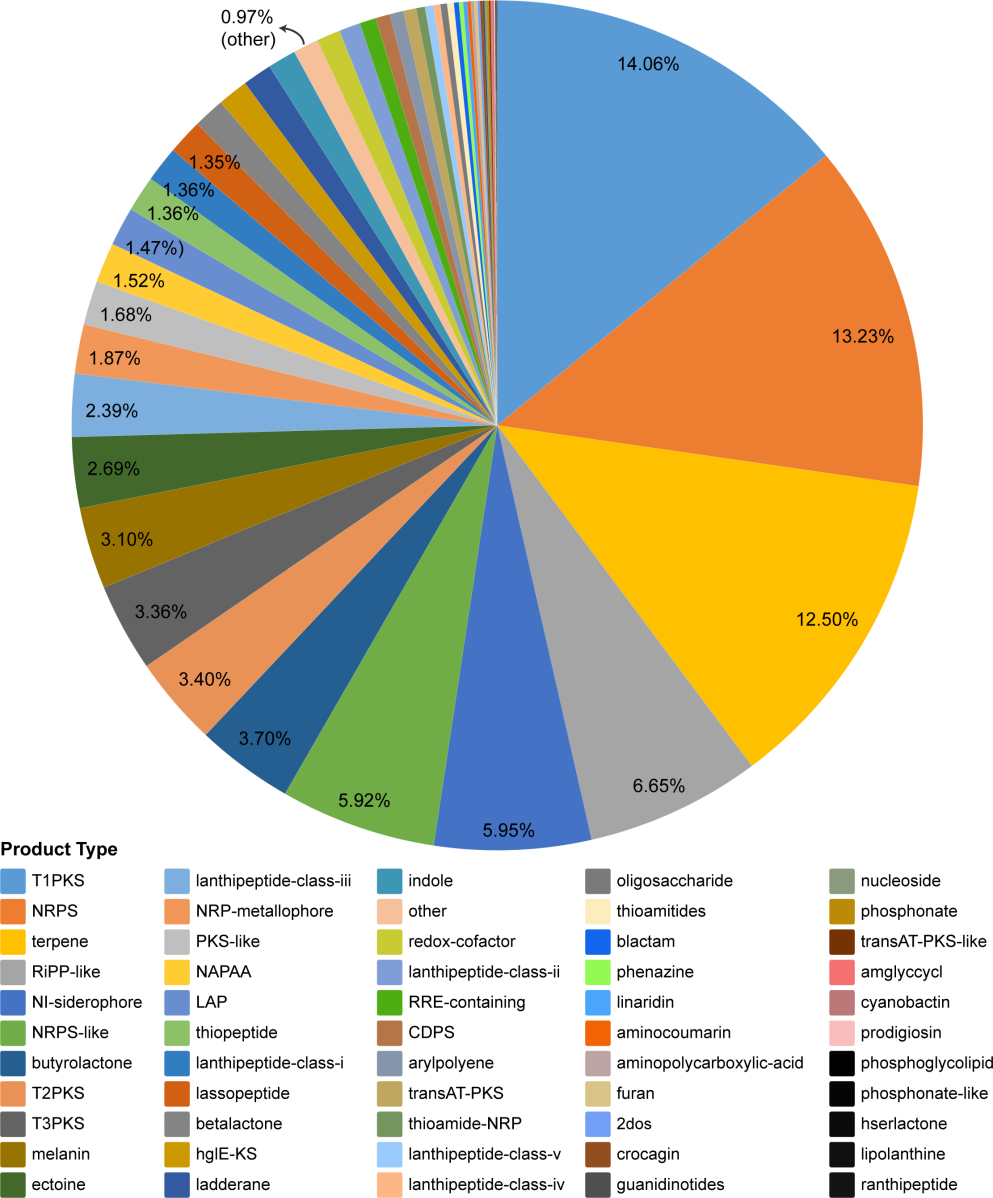
The total count of secondary metabolite types for the 136 *Streptomyces* isolates. Black color represents categories with only one secondary metabolite, and the “other” category (unclassified secondary metabolites) is annotated in the Figure.

To uncover potential *Streptomyces* biosynthetic pathways of bioactive compounds, the SM-BGCs of 136 *Streptomyces* natural isolates were predicted. We counted the total number of each predicted type of secondary metabolite. The results revealed a total of 6,318 products across 55 secondary metabolite types in these *Streptomyces* isolates, with T1PKS, NRPS, and terpene being the predominant classes (Fig. 9). Among them, the least 10 secondary metabolite types were identified as unique or rare (existing in one to four isolates), including phosphoglycolipid, hserlactone, and ranthipeptide, associated with cell-signaling pathways, and the remaining seven types may hold pharmaceutical and industrial development potential (Table S5). For validation of the SM-BGC results from the above bioinformatics analyses, their potential function and bioactivity need more assays, such as thin layer chromatography (TLC), high-performance liquid chromatography (HPLC), liquid chromatography-mass spectrometry (LC-MS), and nuclear magnetic resonance (NMR).

## DISCUSSION

Over 34,000 natural bioactive compounds have been isolated from microorganisms, with 35% originating from Actinobacteria, primarily *Streptomyces* (73). Diverse soil-derived *Streptomyces* often possess novel potential applications and ecological functions worthy of exploration. In this pilot study, we collected 19 soil samples from 14 provinces in China. Using cost-effective library preparation techniques and culture-dependent methods, we undertook a multidimensional exploration into the *Streptomyces* biodiversity in China’s soil. The use of standard population genetic analysis procedures also facilitated detailed exploration of *Streptomyces* genetic diversity. Amplicon analysis revealed *Streptomyces* are present with low relative abundance (0.03% ASVs) in a subset of samples. Using culture-dependent methods, we obtained 136 *Streptomyces* isolates, sequenced and assembled their genomes, and identified rich species diversity (44 species, including 26 unreported). Subsequent population-genomic analysis revealed a high level of genetic diversity among the isolates, correlated with homologous recombination. Pan-genome and secondary metabolite gene cluster analyses provided insights into the genomic diversity, richness of gene clusters, and associated metabolites of the isolated *Streptomyces*.

Compared to traditional studies (18–21) of *Streptomyces* biodiversity, there are some notable improvements in this study. By combining the OSPALC amplicon library construction method (25) with miniaturized genome library preparation techniques (23), a significant reduction in costs was achieved. This has effectively paved the way for conducting large-scale investigations into the biodiversity of *Streptomyces*. In addition, the identification of 26 potentially new *Streptomyces* species highlights significant biodiversity within our isolates. These novel strains may possess unique genomic features (e.g., new genes, gene clusters involved in the synthesis of novel antibiotics or other secondary metabolites, and metabolic pathways for the degradation of specific pollutants) that facilitate the synthesis of bioactive compounds and environmental adaptations (74). Their potential applications in bioremediation and antibiotic production could have notable implications for biotechnology and pharmaceutical industries (75). Importantly, this discovery emphasizes the necessity for further research into *Streptomyces* biodiversity in China, which may uncover previously hidden ecological roles and expand our understanding of microbial functions in soil ecosystems.

Our study found that the isolated *Streptomyces* populations exhibit high genetic diversity, characterized by high π and π_s_ values (Table 1). In addition to effective population size and homologous recombination, factors such as mutation, genetic drift, and natural selection can also contribute to such diversity. Tajima’s D, as another important measure of genetic diversity, indicates that positive values may suggest recent population expansion or the presence of natural selection, while negative values can imply selective pressure, bottleneck effects, or adaptive evolution. Therefore, by analyzing Tajima’s D in conjunction with the distribution of genetic variation (π; π_s_), we can uncover historical events of expansion, contraction, or migration. Furthermore, *F_st_* analysis can detect significantly diverged genes or genomic regions within the isolated *Streptomyces* (Fig. S2), providing a basis for exploring the adaptive functions of these specific genes in future research. Notably, significant *F_st_* values may indicate natural selection targeting specific environments, helping infer the historical and ecological isolation mechanisms of *Streptomyces* populations (76). Overall, integrating genetic parameters with ecological data allows for a comprehensive understanding of the impacts of selective pressure, historical factors, and genomic functions on genetic diversity, which can guide biodiversity conservation strategies and help prioritize the protection of areas with high genetic diversity (77, 78).

Notably, the rich gene diversity revealed by the pan-genome of the 136 *Streptomyces* isolates underscores the presence of unique or rare genes, such as those involved in the production of phosphoglycolipids and hserlactone, which may lead to the synthesis of novel bioactive compounds with potential pharmaceutical and ecological applications. As for the previously analyzed types of these unique biosynthetic gene clusters, future work can focus on characterizing these specific biosynthetic pathways and synthesized compounds to uncover their functions and explore their roles in microbial interactions and environmental adaptations. Understanding how these unique biosynthetic pathways respond to ecological pressures could inform strategies for biotechnological applications, such as drug development and bioremediation (12, 13). In summary, the unique genetic features identified in this study provide a foundation for future research linking genetic diversity to functional diversity, advancing our understanding of *Streptomyces* evolution and its applications.

Nonetheless, our study can be further improved. Although consistent with previous observations indicating a lower abundance of *Streptomyces* compared with other Actinomycetes (64, 65), more ASVs in the amplicon analysis of most soil samples can be obtained by increasing the sequencing throughput. Furthermore, the 16S rRNA gene has an extremely slow evolutionary rate, with approximately 1% nucleotide differentiation every 500,000 years (79), which limits the sensitivity of detecting changes in the *Streptomyces* community structure on a recent evolutionary timescale (ranging from a few thousand to tens of thousands of years) (80). Therefore, caution is advised when using it to describe the diversity of *Streptomyces* “species” or operational taxonomic units (OTUs), as the presence of short sequences, paralogs, or sequencing errors can obscure or inaccurately define their boundaries. Fortunately, the OSPALC amplicon procedures can be easily customized to target virtually any amplifiable gene in the genome, such as the *rpoB* gene, thereby promoting the classification of *Streptomyces* species. The culture-dependent approach in this study also has certain limitations as it might not be able to capture *Streptomyces* isolates that are uncultivable under standard laboratory conditions (81) and the 4-day cultivation period may indeed be insufficient to grow a sufficient number of *Streptomyces* strains to adequately represent their species diversity. Concurrently, the soil or environmental factor is crucial as it poses challenges to the exploration of the mechanisms driving genetic differentiation in *Streptomyces*. Notably, the rapidly advancing field of machine learning may hold promise for an integrative approach to screen a large number of unculturable and diverse *Streptomyces* strains (82, 83).

Through studying the genomes and genetic diversity of *Streptomyces*, we will gain meaningful insights into evolutionary biology and microbial community dynamics, contributing to our understanding of the integral roles that microorganisms perform in evolution and ecosystem functioning. In our study, using recently developed high-throughput methods, we explored the biodiversity of *Streptomyces* in the soil of China from multiple dimensions, unraveling vast species diversity and high levels of genetic diversity, thereby painting a comprehensive picture of *Streptomyces* diversity. Knowledge on the genetic diversity is essential for understanding ecological adaptation, survival, and evolution, as well as facilitating drug development.

## Data Availability

All available raw data in this study, including amplicon and genome Illumina FASTQ files, were uploaded to the NCBI SRA database (BioProject Number: PRJNA1064543). The genome sequences of the 136 Streptomyces isolates are available at Mendeley Data, DOI: https://data.mendeley.com/datasets/gcw2rtxzcb/1.

## SUPPLEMENTAL MATERIAL

Supplemental figures spectrum.01692-24-s0001.docx Figures S1 and S2.

Supplemental tables spectrum.01692-24-s0002.xlsx Tables S1 to S6.

ASM does not own the copyrights to Supplemental Material that may be linked to, or accessed through, an article. The authors have granted ASM a non-exclusive, world-wide license to publish the Supplemental Material files. Please contact the corresponding author directly for reuse.
