# Supplementary material for: Multidimensional insights into the biodiversity of Streptomyces in soils of China: a pilot study
Source: Microbiol Spectr. 2025 Apr 2;13(5):e01692-24. doi: 10.1128/spectrum.01692-24 (PMC12054067; doi:10.1128/spectrum.01692-24)
Supplement: Supplemental figures — Figures S1 and S2. [file spectrum.01692-24-s0001.docx]

**­Supplementary Figures**

**Figures**


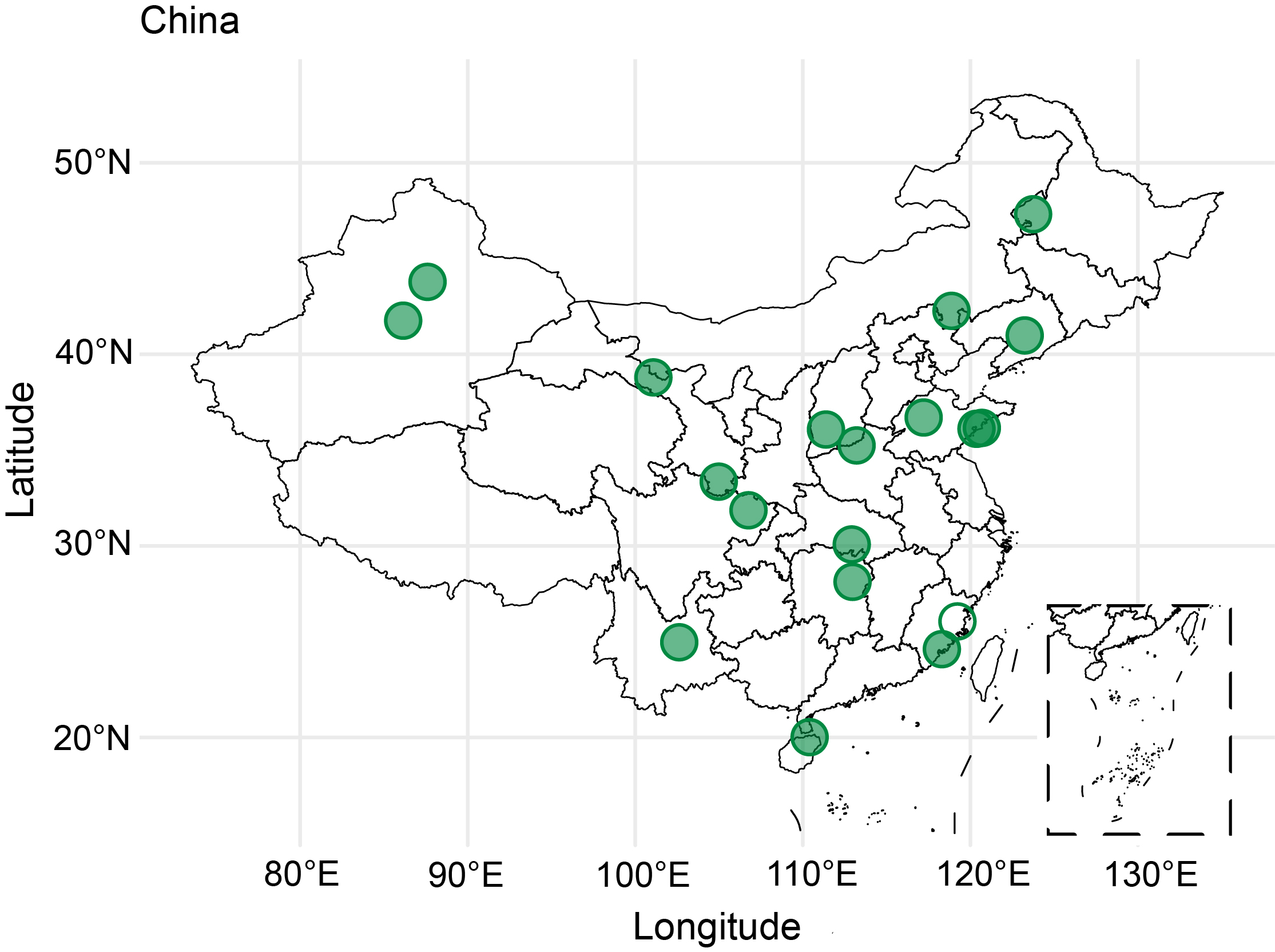


**Fig. S1** Collection sites of 19 soil samples (green bubbles). The unfilled bubble represents the sample for which amplification and sequencing were not successful.


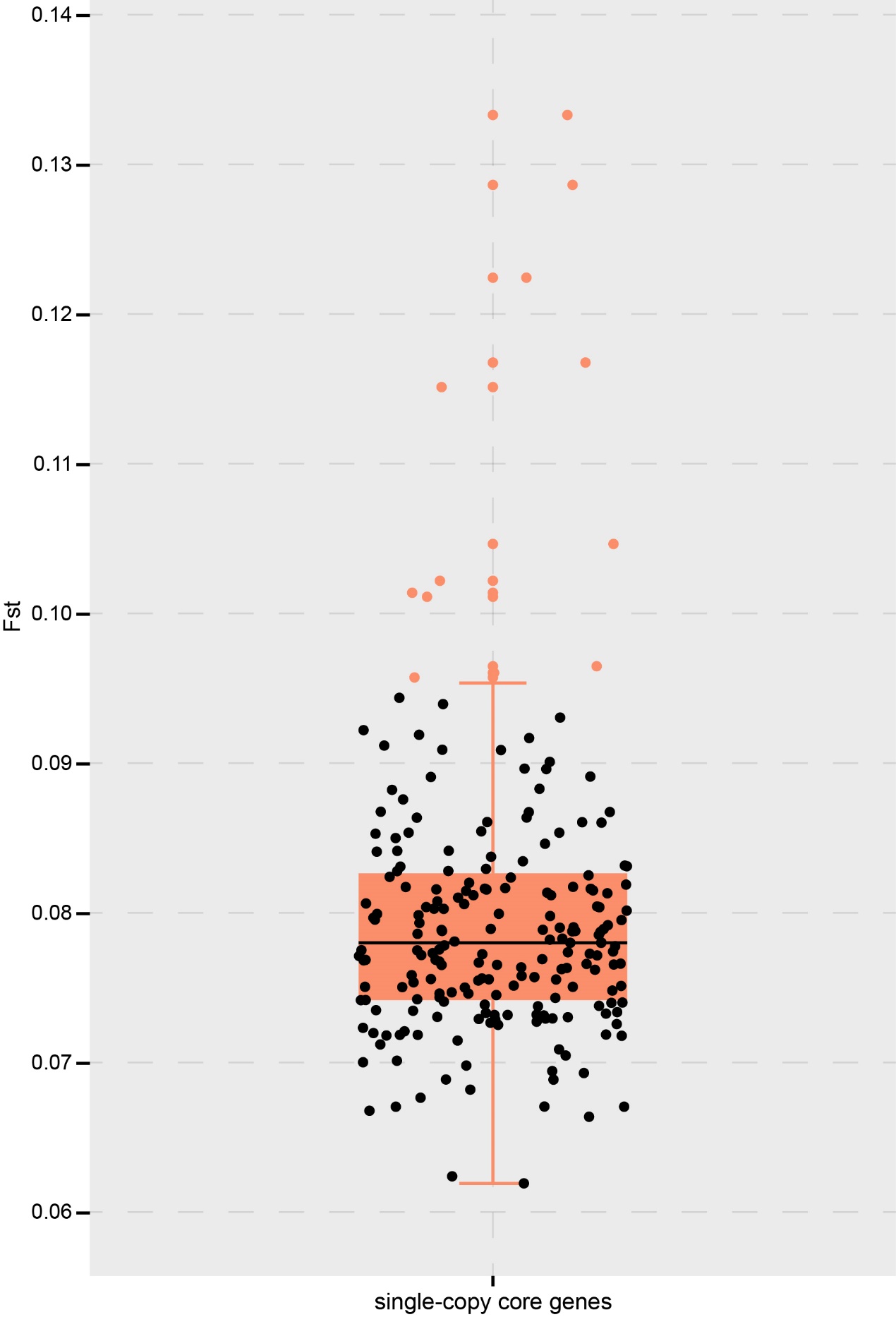


**Fig****. S2** Box plot showing the distribution of Fst values for 215 single-copy genes of isolated *Streptomyces* population, reflecting genetic differentiation. Orange box indicates the median and interquartile range (IQR), while whiskers extend to the minimum and maximum values. Outliers (orange dots) beyond the whiskers represent genes with significant differences in genetic differentiation.
